# Supplementary material for: CODEX: COunterfactual Deep learning for the in silico EXploration of cancer cell line perturbations
Source: Bioinformatics. 2024 Jun 28;40(Suppl 1):i91–9. doi: 10.1093/bioinformatics/btae261 (PMC11211812; doi:10.1093/bioinformatics/btae261)
Supplement: btae261_Supplementary_Data [file btae261_supplementary_data.zip › btae261_Supplementary_Data/Schrod.68.marked.pdf]

Journal Title Here, 2022, pp. 1–9

doi: DOI HERE
Advance Access Publication Date: Day Month Year
Paper

PAPER

CODEX: COunterfactual Deep learning for the *in-silico* EXploration of cancer cell line perturbations

Stefan Schrod,[1,*] Helena U. Zacharias,[2] Tim Beißbarth,[1] Anne-Christin Hauschild[3] and Michael Altenbuchinger[1]

[1]Department of Medical Bioinformatics, University Medical Center Göttingen, Goldschmidtstr. 1, 37077, Niedersachsen, Germany, [2]Peter L. Reichertz Institute for Medical Informatics of TU Braunschweig and Hannover Medical School, Hannover Medical School, 30625 Hannover, Germany and [3]Department of Medical Informatics, University Medical Center Göttingen, Von Siebold Str. 3, 37075, Niedersachsen, Germany

*Corresponding author. stefan.schrod@bioinf.med.uni-goettingen.de

FOR PUBLISHER ONLY Received on Date Month Year; revised on Date Month Year; accepted on Date Month Year

Abstract

Motivation: High-throughput screens (HTS) provide a powerful tool to decipher the causal effects of chemical and genetic perturbations on cancer cell lines. Their ability to evaluate a wide spectrum of interventions, from single drugs to intricate drug combinations and CRISPR-interference, has established them as an invaluable resource for the development of novel therapeutic approaches. Nevertheless, the combinatorial complexity of potential interventions makes a comprehensive exploration intractable. Hence, prioritizing interventions for further experimental investigation becomes of utmost importance.
Results: We propose CODEX as a general framework for the causal modeling of HTS data, linking perturbations to their downstream consequences. CODEX relies on a stringent causal modeling strategy based on counterfactual reasoning. As such, CODEX predicts drug-specific cellular responses, comprising cell survival and molecular alterations, and facilitates the *in-silico* exploration of drug combinations. This is achieved for both bulk and single-cell HTS. We further show that CODEX provides a rationale to explore complex genetic modifications from CRISPR-interference *in silico* in single cells.
Availability and Implementation: Our implementation of CODEX is publicly available at `https://github.com/sschrod/CODEX`. All data used in this article are publicly available.
Supplementary information: Supplementary materials are available at *Bioinformatics* online.

Key words: drug perturbations, causal reasoning, high-throughput screens, deep learning

Introduction

Large-scale perturbation experiments in human cancer cell lines offer a powerful approach to connect genetic or chemical interventions with downstream effects. Such high-throughput screens (HTS) aid the identification of new drug compounds and more effective cancer treatments, and provide a way to study genomic susceptibilities in cancer (Ling *et al.*, 2018).

This motivated several data collections and technical advancements. The Genomics of Drug Sensitivity in Cancer (GDSC) database (Iorio *et al.*, 2016) provides response measurements of 1,001 cancer cell lines to 265 anti-cancer drugs. In comparison to individual drugs, drug combinations can have increased efficacy, and reduced toxicity and adverse side effects as a consequence of reduced dosages (Csermely *et al.*, 2013; Al-Lazikani *et al.*, 2012). This motivated drug combination databases such as DrugComb (Zagidullin *et al.*, 2019), gathering data of more than 700,000 drug combinations for more than 8,000 unique compounds. Investigated downstream effects are not limited to measures of drug response. The Connectivity Map (Subramanian *et al.*, 2017) offers more than 3,000,000 perturbed gene expression profiles using the L1000 technology (measuring the expression of 978 genes) (`https://clue.io/`), containing diverse genetic (shRNA, CRISPR, and overexpression), chemical and physiological perturbations. Advances in barcoding strategies enabled bulk RNA sequencing at comparatively low costs (Bush *et al.*, 2017; Ye *et al.*, 2018) and allowed the generation of perturbation profiles without restricting the gene space (see, e.g., PANACEA (Douglass *et al.*, 2022)). A further essential development are high-throughput single-cell perturbation screens, providing measurements of perturbed transcriptomes of individual cells for diverse interventions. For instance, Sci-Plex was used to screen cancer cell lines exposed to different compounds and dosages at single-cell resolution (Srivatsan *et al.*, 2020). Genetic perturbations on a single-cell level were approached by Perturb-Seq, which uses barcoding techniques and CRISPR interference to perform genome-scale perturbation screens covering more than 1,000 gene knockouts on RPE-1 and K562 cells (Replogle

© The Author 2022. Published by Oxford University Press. All rights reserved. For permissions, please e-mail: journals.permissions@oup.com

et al., 2022). Further, Norman *et al.* (2019) explored the effects of 131 two-gene knockouts on K562 cells. Even though more than 100,000 single cells are recorded, only a small fraction of the combinatorial space could be experimentally covered, highlighting the need for computational approaches to infer novel drug combination effects and guide experimental studies.

The outlined resources differ with respect to the employed techniques, and the investigated downstream effects and interventions. However, different data types typically require tailored solutions. For instance, drug sensitivity predictions in cell lines were addressed in an NCI-DREAM challenge (Costello *et al.*, 2014) and identified a Bayesian multitask multiple kernel learning approach to perform best. Subsequent methods built on this idea and additionally addressed the model adaptation to real tumor specimens to provide treatment response predictions in individual patients (Sharifi-Noghabi *et al.*, 2021; He *et al.*, 2022). The prediction of drug synergies was pioneered by DeepSynergy (Preuer *et al.*, 2018). DeepSynergy predicts drug synergisms from cell-line transcriptomic data in combination with features representing compound structures. Drug response predictions in single cells were addressed by the Compositional Perturbation Autoencoder (CPA) (Lotfollahi *et al.*, 2023), which also addressed drug combinations, by perturbing the latent representation of a Variational Autoencoder (VAE). The prediction of unseen genetic interventions and combinations thereof was addressed by GEARS (Roohani *et al.*, 2023). GEARS utilizes Graph Neural Networks (GNNs) to incorporate prior knowledge of gene-gene relationships into the model architecture. The generative approach, however, limits applications to a specific cell type. In summary, all outlined approaches are tailored to the problem at hand, even though they describe the common problem of predicting causal consequences of a specified intervention.

The inference of many causal actions necessitates counterfactual reasoning. For instance, in a typical clinical trial, patient outcome is recorded for a treated and a control group. By comparing the outcomes of both groups, the average treatment effect can be derived. However, a treatment that is beneficial on average may not be helpful for every individual patient. The computational challenge in predicting individual patients' treatment responses arises from the fact that patients' outcomes can only be observed for the specific treatment they received, not for the alternative treatments they did not receive (here, the control) (Rubin, 1974). Consequently, algorithms cannot directly learn rules for selecting the "better" treatment for each patient. Instead, they must infer the alternative, or "counterfactual", outcomes in order to adequately assess the treatment's relative benefit. In HTS, alternative interventions can be observed, assuming that cultures of the same cell line can be considered as technical replicates. Nevertheless, it is impractical to test every possible intervention, especially for combinations of interventions; the combinatorial complexity makes a comprehensive exploration intractable. Thus, *in-silico* approaches become necessary to prioritize interventions for further experimental investigations. To address this task, we will build on counterfactual machine learning approaches to extrapolate the space of interventions to the yet unseen "counterfactual" perturbations and cell lines.

Counterfactual deep-learning (DL) approaches turned out to be particularly promising due to their enormous flexibility. The typical strategy is to construct networks which facilitate joint representation learning across all investigated interventions, and to account for treatment specific effects via dedicated network branches (Johansson *et al.*, 2016; Shalit *et al.*, 2017; Yao *et al.*, 2018; Schrod *et al.*, 2023). In the context of HTS data, molecular information is first aggregated in a treatment-agnostic manner to encode features of unperturbed control cells and then separated into treatment-specific representations to capture the treatment underlying molecular mechanisms. Note that a dedicated representation has to be trained for each intervention or intervention combination, and as such, downstream effects of novel combinations cannot be inferred. Alternative approaches that only consider the intervention as a predictor variable typically capture only average treatment effects and may overlook individual treatment effects. This can be explicitly seen from the comparison of an ordinary regression model, which uses a treatment variable, and a T-learner, which consists of a set of individual treatment-specific models (Shalit *et al.*, 2017; Schrod *et al.*, 2023). Notably, most recent counterfactual DL approaches also take into account potential treatment selection biases as might be present in observational studies, for which regularization techniques for distributional balancing are used or adversarial learning techniques (Johansson *et al.*, 2016; Shalit *et al.*, 2017; Yao *et al.*, 2018; Yoon *et al.*, 2018; Schrod *et al.*, 2022). Confounding effects due to treatment biases, however, might be of less relevance in the context of HTS, where interventions are *a priori* unbiased.

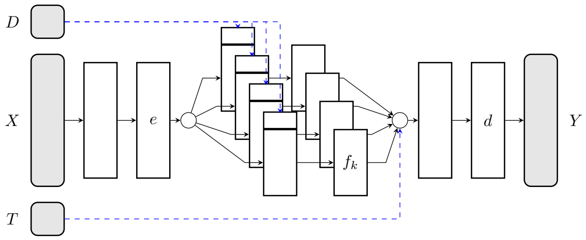

Fig. 1. CODEX architecture used for causal representation learning: an unperturbed profile X is transformed to map the effect of specific interventions T, which might be associated with a dosage D, to the perturbed outcome Y. The unperturbed state is first encoded in a latent state e, which is passed through the respective treatment specific representations f_t. Treatment combinations are naturally incorporated by simultaneously propagating profiles through respective treatment arms. Further, if dosage specific interventions are used, the dosage is incorporated as input variable to the intervention specific layers. Finally, the individual effects are aggregated and combined by a shared decoder d to model the perturbed outcome.

Here, we will introduce CODEX (COunterfactual Deep learning for the *in-silico* EXploration of cancer cell line perturbations), which builds on counterfactual DL approaches to provide a general framework to model HTS data. In contrast to existing counterfactual DL approaches, CODEX facilitates the prediction of unseen perturbation combinations by learning from individually applied interventions and complementary combinations. CODEX can account for non-linear combinatorial effects and can incorporate prior knowledge about gene-gene relationships, such as provided by Gene Ontologies (GO). We demonstrate that CODEX can extrapolate the space of interventions to new cell lines and new treatment combinations, and via prior knowledge even to completely unseen perturbations and combinations thereof. This is illustrated for both bulk and single-cell transcriptomics data, and for the predictions of drug responses and perturbed gene-expression profiles.

Material and Methods

CODEX

Model architecture

The CODEX approach (Fig. 1) is based on deep neural network architectures for counterfactual reasoning (Johansson *et al.*, 2016; Shalit *et al.*, 2017; Yoon *et al.*, 2018; Schrod *et al.*, 2022). Let $\mathbf{x}_i$ represent a vector of unperturbed molecular features that characterizes a bulk cell line specimen i, or, in the context of single-cell HTS, an unperturbed single cell i. Throughout our analyses, gene expression levels are used as input features, but in principle, any omics data could be used. Note, however, that transcriptomics data are most readily available and have been shown to be most relevant for drug sensitivity predictions (Costello *et al.*, 2014). Further, let the vector $\mathbf{t}_i = (t_i^1, \ldots, t_i^K)$ represent the perturbations applied in experiment i, where each element $t_i^k \in 0, 1$ indicates whether intervention k was applied (1) or not (0). Thus, $\mathbf{t}_i$ encodes the complete set of perturbations applied in experiment i. In case of chemical interventions, an additional vector $\mathbf{d}_i = (d_i^1, \ldots, d_i^K)$ encodes drug dosages, respectively. The prediction target will be denoted as $\mathbf{y}_i \in \mathcal{Y}$, which can be any variable or set of variables characterizing the outcome of the perturbation, such as measures of drug efficacy (Costello *et al.*, 2014), drug synergies (Preuer *et al.*, 2018), as well as perturbation profiles (Lotfollahi *et al.*, 2023; Roohani *et al.*, 2023). Thus, CODEX requires the triplet of input information consisting of a control (the unperturbed transcriptome), the intervention (potentially associated with dosages), and the recorded downstream effects.

Causal inference models such as (Shalit *et al.*, 2017) do not incorporate the action as a covariate but rather as a structural parameter in order to train intervention specific data representations. CODEX builds on this concept. Specifically, we first train a latent shared embedding of the initial state $e : \mathbb{X} \to \mathbb{R}^d$ to reduce the dimensionality of the problem and to construct prediction-relevant features. This embedding is then passed through intervention specific layers (a mapping $f_k : \mathbb{R}^d \to \mathbb{R}^d$) to account for the molecular downstream effects induced by intervention k. Importantly, we do not introduce individual representations for combinations of treatments. While this would be a direct adaptation of counterfactual DL models like those of (Shalit *et al.*, 2017), it would significantly inflate the parameter space and hinder the inference of unseen combinations. This is because each treatment combination would require observations to train the respective network branch and to facilitate any kind of predictions, making existing causal inference models impracticable for complex HTS data. Thus, we pass the latent variables $e(X)$ through a set of active network branches corresponding to the respective single interventions. Subsequently, they are passed through additional joint layers – a decoder $d : \mathbb{R}^d \to \mathbb{Y}$ – to reconstruct the downstream effects. In case of multiple simultaneously applied interventions, the corresponding latent representations are aggregated before the combinatorial effect is deciphered by the decoder. One should note that, even though the effects are linearly aggregated on a latent state of the model, the decoder naturally captures non-linear combinatorial effects. In summary, the CODEX mapping reads:

$$\hat{y}_i = d\left(\sum_{j=1}^{K} t_i^{(j)} f_j(e(\mathbf{x}_i))\right) , \qquad (1)$$

which implicitly sums all active treatment branches associated with non-zero coefficients $t_i^{(j)}$. Note that inactive treatment branches $t_i^{(j)} = 0$ are not explicitly evaluated to save computational resources.

CODEX for drug-synergy prediction

CODEX is a general architecture to model causal effects and their combinations, and as such, enables predictions of diverse outcome measures. In this work, we focus on the prediction of drug synergies and the reconstruction of perturbed gene expression profiles. Drug synergies are commonly expressed in terms of synergy scores $Y \in \mathbb{R}$, which quantify the difference between experimentally tested response surfaces and theoretical models combining individual treatments naively, such as Loewe Additivity (Loewe, 1953) or Bliss independence (Bliss, 1939). In this work, we focus on the Zero Interaction Potency (ZIP) score, which takes advantage of both the Loewe Additivity and the Bliss independence model (Yadav *et al.*, 2015). To model drug synergies via CODEX, we map the decoder to a Mean Squared Error (MSE) loss

$$\min_{\Theta} \frac{1}{N} \sum_i (\hat{y}_i - y_i)^2 , \qquad (2)$$

where the parameter space is given by $\Theta = [\theta_e, \theta_{f_t}, \theta_d]$. The applied interventions are implicit in Eq. (1). The full model architecture and the hyper-parameter search space are given in the Supplementary Materials.

CODEX for the prediction of single-cell perturbation profiles

Synergy scores are summary statistics of potentially complex molecular downstream effects. In recent years, downstream effects have become increasingly available in terms of perturbed transcriptomes in both bulks and single cells (Subramanian *et al.*, 2017). We focus on the latter with $y_i = (y_{i1}, \ldots, y_{ip}) \in \mathbb{R}^p$ corresponding to a perturbed transcriptome with y_{ij} representing the expression of gene j in cell i. Let $z_i = \sum_{j=1}^{K} t_i^{(j)} f_j(e(\mathbf{x}_i))$, which is the linear combination of the latent variables representing active treatment branches in cell i. CODEX then uses a Gaussian loss which quantifies the accuracy of predictions of y_i together with respective gene variances,

$$\min_{\Theta} \sum_{i,j} 1/2 \left(\log(d_{\sigma^2}(z_i)_j) + \frac{(d_\mu(z_i) - y_i)_j^2}{d_{\sigma^2}(z_i)_j} \right) , \qquad (3)$$

where $d_\mu(z_i)_j$ maps to the mean expression of gene j and $d_{\sigma^2}(z_i)_j$ infers the variance of the estimates, given the respective treatment or treatment combination. We further imposed that $d_{\sigma^2}(z_i)_j$ is larger than $\epsilon = 10^{-6}$ to increase numerical stability.

CODEX for the prediction of drug dosage effects

Different drug dosages add to the combinatorial complexity of intervention experiments and can introduce complex non-linear dependencies. In principle, dosage effects can be incorporated into CODEX via different treatment branches. This, however, would prohibit the extrapolation to unseen drug dosages and would substantially increase model complexity. Therefore, rather than introducing additional dose representations, CODEX incorporates dosage information as an additional categorical feature d_i^k which adds to the first layer of each treatment specific branch k (Fig. 1).

Table 1. Mean 5-fold cross-validation performance for the prediction of ZIP synergy scores, in terms of Spearman Correlation Coefficient (SCC), Pearson Correlation Coefficient (PCC) and Mean Squared Error (MSE) for unseen cell lines (1) and unseen drug combinations (2). The best performing scores were highlighted in bold, and the arrow indicates whether the measure is maximized or minimized.

Model	Setting (1)			Setting (2)		
	SCC ↑	PCC ↑	RMSE ↓	SCC ↑	PCC ↑	RMSE ↓
CODEX	0.752 (±.004)	**0.889 (±.005)**	**5.31 (±.18)**	0.725 (±.005)	**0.877 (±.005)**	**5.58 (±.17)**
MARSY	**0.780 (±.010)**	0.886 (±.005)	5.36 (±.19)	**0.749 (±.011)**	0.875 (±.005)	5.62 (±.15)
MatchMaker	0.742 (±.004)	0.873 (±.006)	6.11 (±.32)	0.720 (±.006)	0.864 (±.007)	6.23 (±.12)
TreeCombo	0.737 (±.004)	0.870 (±.003)	5.73 (±.17)	0.689 (±.005)	0.856 (±.006)	6.00 (±.15)
DeepSynergy	0.701 (±.003)	0.869 (±.004)	5.78 (±.18)	0.676 (±.006)	0.860 (±.008)	5.95 (±.16)

CODEX for the prediction of unobserved perturbations

CODEX infers a distinct model branch for each individual perturbation, effectively reducing the number of model representations to the number of distinct perturbations. However, it does not require that those perturbations were independently observed. Rather, the training data can comprise perturbations observed in combinations. Consequently, each combination adds information to decipher the individual model branches. We make use of this feature and introduce a weighting scheme to share information among perturbations.

We illustrate this concept for CRISPR interference screens, where the individual perturbations correspond to silenced genes. In this case, we use gene similarities derived by (Roohani *et al.*, 2023), which aggregate information from GO (Consortium, 2004). The basic idea is to compute the Jaccard index between a pair of genes j,j' as $\mathcal{J}_{j,j'} = \frac{|N_j \cap N_{j'}|}{|N_j \cup N_{j'}|}$, where N_u is the set of pathways containing gene u. This is the fraction of shared pathways between two genes.

Consider an unobserved gene knockout of gene j'. Then, we can construct the treatment proxy model by setting $t_i^{(j)} = C\mathcal{J}_{j,j'}$, where C is determined by normalization $C = \sum_{j=1}^{K} t_i^{(j)}$. In the case of single unobserved perturbations, the proxy vector is used as is, otherwise, respective proxy vectors and observed treatment vectors are added up. For instance, let $(1,0,0,0,0)$ be a treatment vector, where the observed interventions are indicated by the 1 at the first position. Further, let $(0,0.4,0.35,0.25,0)$ be a normalized proxy treatment vector, determined as outlined. Then, we evaluated CODEX with the sum of both, $\mathbf{t}_i = (1,0.4,0.35,0.25,0)$. Thus, in summary, CODEX passes the data through treatment branches which likely resemble the unobserved perturbation. It is noteworthy that the proposed weighting scheme is one specific choice to incorporate prior knowledge to share information among perturbations. Other, viable options could rely, e.g., on measurements of protein-protein interactions or sequential similarity.

For more details about the implementation and the used network architecture refer to the Supplementary Materials.

Competing methods

Algorithms for drug-synergy predictions in cell lines

We compared CODEX to

- TreeCombo (Janizek *et al.*, 2018), which is a tree-based approach using Extreme Gradient Boosting (XGBoost),
- DeepSynergy (Preuer *et al.*, 2018), a dense feed forward neural network to predict synergy scores,
- MatchMaker (Kuru *et al.*, 2021), which is inspired by DeepSynergy but additionally splits the network into two parts representing the two different interventions, and
- MARSY (El Khili *et al.*, 2023), which uses a drug-drug representation network combined with the latent representation learner of DeepSynergy.

In contrast to CODEX, the competing models additionally use chemical drug encodings as input.

Algorithms for the prediction of post-perturbation profiles

We compared CODEX to:

- Random Baseline: in line with Lotfollahi *et al.* (2023), we implemented a random baseline to assess the relative benefit of CODEX in the context of dosage extrapolations.
- Linear baseline: we implemented a linear baseline which simply averages the downstream predictions of individual perturbations to predict the effect of perturbation combinations.
- Gene Regulatory Network (GRN): GRN, as implemented by Roohani *et al.* (2023), infers a GRN to linearly propagate the effect of gene perturbations.
- Compositional Autoencoder (CPA) (Lotfollahi *et al.*, 2023): CPA is based on a Variational Autoencoder (VAE) architecture trained to encode both control and perturbation profiles. It encodes different perturbations and dosages in a latent space. This space is made indistinguishable with respect to the different interventions using an adversarial discriminator. Downstream effects are predicted by (1) encoding control cells and (2) decoding them with respective interventions activated in the latent space.
- Graph-Enhanced gene Activation and Repression Simulator (GEARS) (Roohani *et al.*, 2023): GEARS is a generative approach assuming a single type of cell. It uses two separate Graph Neural Networks (GNNs) to encode additional prior knowledge about gene-gene relationships and perturbation relationships. The first GNN embeds the unperturbed state using a gene co-expression knowledge graph, and the second GNN learns perturbation embeddings using a graph derived from GO. The states are combined using the respective perturbations, and a feed-forward decoder is used to reconstruct the post-perturbation gene expression.
- Linear CODEX: lin-CODEX removes the non-linear effect combination from CODEX. This baseline is included to illustrate the benefit of the effect decoder and thus serves as an ablation study. For model inference, the trained CODEX model is evaluated only for individual model branches and subsequently averaged to receive predictions for respective treatment combinations.

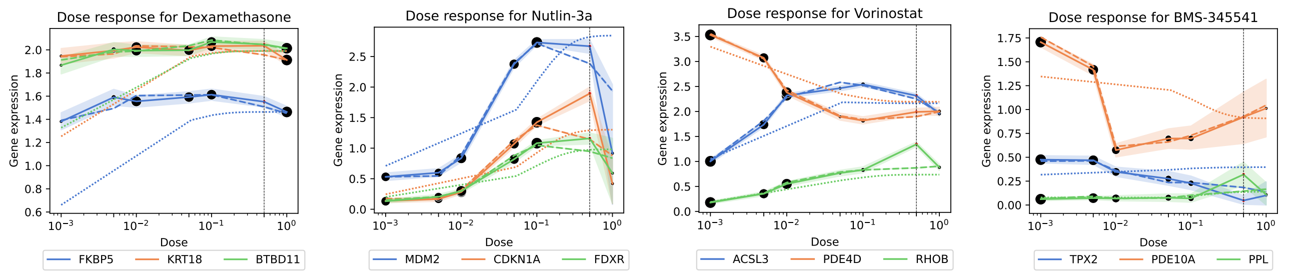

Fig. 2. Dose-dependent reconstruction of the gene expression levels of the top 3 DEGs for each of the treatments. Ground truth values (solid lines and error bars), with the size of the dots representing the number of available samples for each measurement, are contrasted with predictions by CPA (dotted lines) and CODEX (dashed lines). The dashed vertical line indicates the dosage left out for testing.

Results

CODEX improves drug-synergy predictions in cancer cell lines

DrugComb (Zagidullin *et al.*, 2019) provides more than 700,000 recorded drug combinations for 8,379 different drugs on 2,320 different tissues, making it an invaluable resource to explore drug combinations. However, the combinatorial complexity prohibits a comprehensive experimental exploration and asks for computational solutions. For instance, exploring all combinations of two drugs in all tissues would correspond to $\sim 10^{11}$ experiments. Following El Khili *et al.* (2023), we extracted 670 unique drugs and a set of 2,353 corresponding drug pairs on 75 selected cancer cell lines. Synergy effects were extracted from DrugComb (Zagidullin *et al.*, 2019), and the normalized untreated cancer cell lines were retrieved from CellMiner (Reinhold *et al.*, 2012). Lowly expressed genes with $\log_2(\text{RPKM}+1) < 1$ and a variance smaller than 0.8 were excluded, resulting in a final set of 4,639 features. For validation, we followed El Khili *et al.* (2023), and performed two different settings:

(1) a 5-fold cross-validation, where random triplets of cell line-drug-drug combinations were selected for testing,
(2) a stratified 5-fold cross-validation strategy, where individual treatment combinations were removed from the training, meaning that the test fold contains unseen drug combinations.

This validation strategy does not prohibit that similar cell lines and perturbations are captured by the training and test data, which enables meaningful extrapolations among cell lines and perturbations. For performance comparison, we evaluated the Zero Interaction Potency (ZIP) (Yadav *et al.*, 2015), which measures the change in potency of the dose-response curves for individual drugs and their combinations. The main assumption is that non-interacting drugs do not change the underlying dose-response curves. ZIP combines the Bliss independence model (Bliss, 1939) and Loewes Additivity (Loewe, 1953), using fitted dose-response curves rather than experimentally observed points. All baselines were extracted from (El Khili *et al.*, 2023).

Performance comparison

The results are given in Table 1. We evaluated three performance measures: the Spearman Correlation Coefficient (SCC), the Pearson Correlation Coefficient (PCC) and the MSE between ground truth and predictions. In the first scenario, the test set contained drug-drug combinations which were already part of the training data, although they were not seen in the same cell line. CODEX yielded the highest PCC and lowest MSEs between predictions and ground truth values and was only out-competed by MARSY with respect to SCC. The remaining competitors performed substantially worse than both approaches.

Next, we evaluated the more challenging scenario of validation experiment (2), where the test fold contains only unseen drug combinations. As expected, all approaches performed worse than in the validation experiment (1). However, both CODEX and MARSY still achieve reasonable performance with PCCs $>$ 0.87. As previously, CODEX performed best with respect to PCC and MSE and MARSY with respect to SCC. The same holds true considering an alternative synergy score S_{mean}, where MARSY slightly out-competed CODEX, while the other approaches showed inferior performance (refer to Supplementary Table S1).

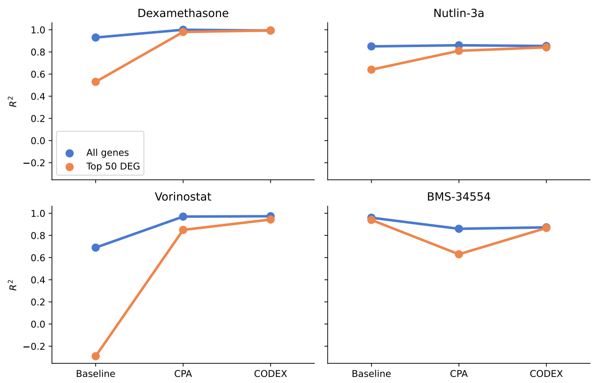

Fig. 3. R^2 reconstruction performance of the mean post perturbation gene expression of all genes (blue) and the top 50 DEGs (orange) obtained for the second highest dose (left out for testing) on Sciplex2.

CODEX improves dose-response predictions in single-cell data

We studied CODEX's ability to predict dose specific responses in single-cell perturbation data. We used the Sciplex2 data (Srivatsan *et al.*, 2020), which contain 12,656 post-perturbation transcriptomic profiles of A549 human lung adenocarcinoma cells measured for 4 different perturbations (Vorinostat, BMS-34554, Dexamethasone and Nutlin3a) and 7 different concentrations (in total 28 drug-dose combinations). We used the preprocessed data provided by Lotfollahi *et al.* (2023), which were normalized and log transformed and limited

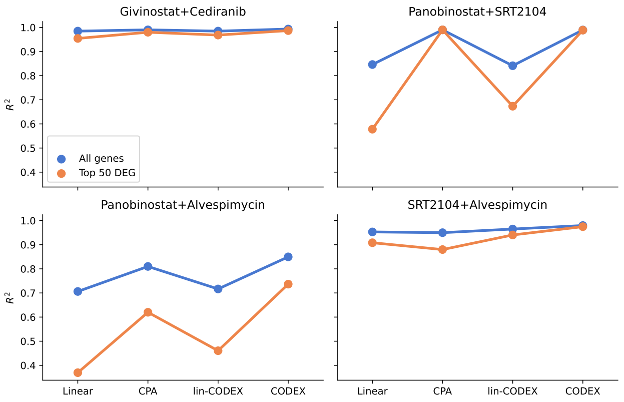

Fig. 4. R^2 reconstruction performance of the mean post perturbation gene expression profiles for all genes (blue) and top 50 DEGs (orange) obtained for the four held out treatment combinations on the Combosciplex data.

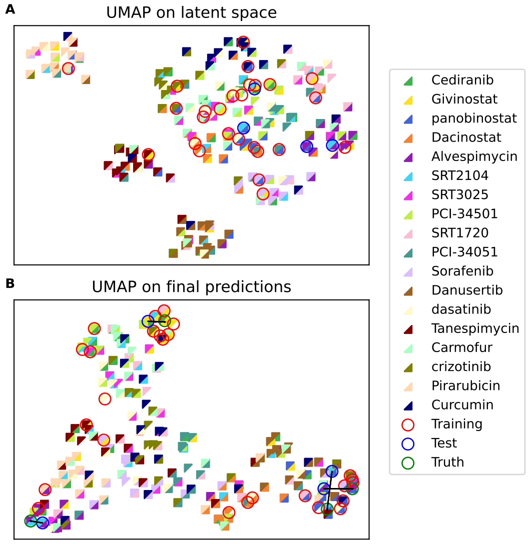

Fig. 5. UMAP of the combined latent representation z of CODEX (A) and of its final predictions (B) for all possible treatment combinations of the Combosciplex data. Each colored triangle represents a treatment component, and the full squares represent their respective combinations. Training combinations are circled in red, test combinations in blue, and the ground truth for the unobserved combinations in green (only for final predictions).

to the 5,000 most variable genes. Accordingly, we left out the second highest treatment concentration and tested CODEX ability to reproduce the dose-response curves and to interpolate to unseen concentrations.

Performance comparison

Fig. 2 shows dose-response curves (response versus dosage) for the top three Differentially Expressed Genes (DEGs) for each treatment. Ground truth values (solid lines and error bars), with dots representing individual measurements, are contrasted with predictions by CPA (dotted lines) and CODEX (dashed lines). The second highest dosage was hold out for testing and is highlighted by a vertical dashed line. CODEX achieves a substantially better description of both training and test dosages, capturing also non-linear dependencies (Fig. 2). This is also supported by considering the reconstruction performance in terms of the coefficient of determination R^2 (Fig. 3), for all genes (blue) and for the top 50 DEGs (orange). CODEX performed best for Dexamethasone, Nutlin-3a, Vorinostat and the linear baseline for BMS-34554, where substantial improvements on the top 50 DEGs were observed for the drugs Vorinostat (0.94 vs. 0.85 for CODEX vs. CPA), and Nutlin-3a (0.84 vs. 0.81 for CODEX vs. CPA). On average, we observed a median performance gain of 9.1% for the top 50 DEGs compared to CPA (Supplementary Fig. S1A). Furthermore, considering all dosages (both validation and out-of-distribution data), CODEX showed significantly improved R^2 values compared to CPA, and a median performance increase of 11.5% for the top 50 DEGs (Supplementary Fig. S1B). We further observed that the linear baseline performs well on BMS-34554, while it failed for Dexamethasone and Vorinostat. This suggests that linear effects are sufficient to model some but not all interventions.

CODEX can predict molecular downstream effects of drug combinations in single cells

We further investigated molecular downstream effects in terms of perturbed transcriptomes of 13 anticancer drugs on A549 cells obtained from the combinatorial sci-Plex (Combosciplex) assay (Lotfollahi *et al.*, 2023). The data comprise 18 different anti-cancer medications evaluated on 63,430 single cells, including a total of 25 unique drug combinations and 7 individually observed drug perturbations (for an overview, see Supplementary Table S5). Similar to the Sciplex2 data, Combosciplex was normalized, log-transformed, and restricted to the 5,000 most variable genes. To study the ability of CODEX to infer the effects of unseen drug combinations, we excluded four combinations during model development and evaluated the reconstruction of perturbed transcriptomes using R^2 scores. We compared CODEX to the linear baseline, which averages the observed single effects, CPA (Lotfollahi *et al.*, 2023), and lin-CODEX.

Performance comparison

Considering median R^2 values, we observed that CODEX performs best with an increase of 5.5% on the top 50 DEGs compared to CPA (Supplementary Fig. S2). The performance resolved for the individual hold-out treatment combinations is shown in Fig. 4. There, already lin-CODEX is able to improve predictions compared to the linear baseline. However, it is outperformed by both CPA and CODEX, suggesting a crucial role of non-linear decodings (see also Supplementary Fig. S2). The performance gains of CODEX were mainly attributed to the combinations including Alvespimicin (Fig. 4). Those were weakly supported by the training data with significantly different effects than observed during training (Lotfollahi *et al.*, 2023). This is further illustrated in a UMAP representation, where the left-out treatment combinations containing Alvespimin (purple triangles, with blue circles) are separated from the majority of the training combinations (red circles), both on the latent space and the final predictions (Fig. 5A and B, see also Supplementary Fig. S3 for increased resolution).

The Combosciplex data comprise only a subset of all possible drug combinations. We next used CODEX to infer all remaining unobserved drug combinations and visualized the results using a UMAP representation on the latent linear effects (z_i) and on the final predictions (top and bottom of Fig. 5, respectively).

UMAP on the latent representation reveals distinct clusters associated with the dominant effects attributed to Pirarubicin, Danusertib, Tanespimycin, and Sorafenib. Those are not revealed on the final predictions (bottom Fig. 5), suggesting that the non-linear adjustments of the decoder regulate the treatment effect sizes. We further confirmed that out-of-sample predictions (blue circles) are in near vicinity of respective ground truths (green circles), with one-to-one correspondence indicated by connecting black lines.

CODEX facilitates predictions of combined gene knock-out perturbations from CRISPRi in single cells

Finally, we used CODEX to explore genetic perturbations implemented via CRISPRi. CRISPRi facilitates the targeted silencing of genes and has become increasingly feasible in large-scale single-cell perturbation screens in recent years. For instance, Norman *et al.* (2019) proposed Perturb-seq to perform single-cell pooled CRISPRi screens and provided data containing a total of 284 unique knock-out conditions, comprising 131 unique two-gene knockouts, on 108,000 single-cells from the K562 cancer cell line (Norman *et al.*, 2019). In the first experiment, we selected test combinations where the individual perturbations were part of the training data. To further guarantee a fair comparison to state-of-the-art competitors, we followed Roohani *et al.* (2023) and implemented the same 5 test-training splits using the same feature set of 5,045 genes. We assessed the performance in reconstructing perturbed profiles in terms of normalized MSE on the top 20 DEGs (normalized using the reconstruction error of the random baseline) and PCC, where we evaluated the reconstructed effect on all genes (not considering the control background) and the reconstruction of the top 20 DEGs.

Performance comparisons

Also, considering this application, CODEX substantially improves the recently suggested state-of-the-art solutions CPA and GEARS and by far out-competes GRN. Considering PCC for the top 20 DEGs (Fig. 6C), we observed a median value of 0.98 for CODEX compared to 0.96 for lin-CODEX, 0.91 for CPA, 0.92 for GEARS, and 0.82 for GRN. This is consistent with the performance in terms of normalized MSEs (Fig. 6A) and by considering PCC for all genes (Fig. 6B). To further substantiate these findings, we performed an additional validation experiment corresponding to the setting of (Lotfollahi *et al.*, 2023), where each combination of perturbations is left out in one of 13 splits, and evaluated the reconstruction performance in terms of the R^2 score on all genes and on the top 50 DEGs, in line with (Lotfollahi *et al.*, 2023). These results demonstrate that in this comparison, CODEX out-competes the other methods significantly (Supplementary Fig. S4).

CODEX facilitates predictions of unobserved gene knock-outs from CRISPRi in single cells

CODEX can predict downstream effects of unseen perturbations, combinations thereof with observed perturbations, as well as combinations of unseen perturbations only. This is achieved by proxy models resembling unseen perturbations. These proxies were established by a weighting scheme summarizing related gene perturbations (see Methods). To assess CODEX's capability to predict unseen perturbations, we performed three additional experiments using the Norman *et al.* (2019) dataset, where we inferred the effect of left-out perturbations for a

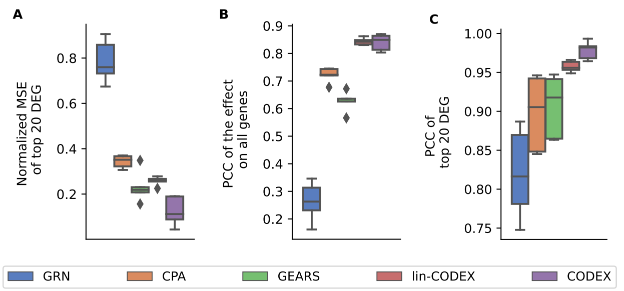

Fig. 6. Reconstruction performance of unseen perturbation combinations on the Norman *et al.* (2019) data.

single missing perturbation (0/1 seen), a missing perturbation in a pair (1/2 seen), and two missing perturbations in a pair (0/2 seen). Due to the limited number of single gene perturbations (105), we tested CODEX on two additional genetic perturbation screens generated by Replogle *et al.* (2022), comprising a total of 1,543 RPE-1 and 1,092 K562 single genetic perturbations, with 175,398 and 192,648 measured single cells, respectively. We again adapted the experimental setup of Roohani *et al.* (2023) and compared the reconstruction error based on 5 identical sets of held-out perturbations. Performance was again assessed using normalized MSE on the top 20 DEGs, PCC of the effect on all genes, and PCC on the top 20 genes.

Performance comparison

For the Norman *et al.* (2019) data, we observed that GEARS yielded the lowest median MSE in all three settings (Fig. 7A). However, CODEX consistently out-competes all other methods with respect to PCC (Fig. 7B and C). There, CODEX improves the median PCC on the top 20 DEGs from 0.90 to 0.92 for 0/1 seen, 0.83 to 0.93 for 1/2 seen, and 0.79 to 0.89 for 0/2 seen compared to the second best performing model (GEARS).

On the Replogle *et al.* (2022) data, CODEX performs best on all compared measures for both K562 and RPE-1 cells (Fig. 8a-c). On K569 cells, CODEX improves the median PCC from 0.31 to 0.49, and on RPE-1 cells from 0.53 to 0.61, compared to GEARS (Fig. 8b). In comparison, the performance of CPA and GRN is highly compromised with respect to MSE (Fig. 8a) and PCC on all genes (Fig. 8b). These findings suggest that when many single perturbations are known, gene graph proxies can be highly efficient. This holds true for both CODEX and GEARS. For CODEX, however, this aspect seems to be even more beneficial.

Conclusion

We proposed CODEX as a general framework to model high-throughput perturbation experiments. CODEX naturally facilitates diverse causal prediction tasks, can learn non-linear effect combinations, and can model different intervention types. This was shown for both chemical and genetic perturbation combinations. Moreover, we suggested a weighting scheme to perform predictions for completely unseen perturbations. Thus, applications of CODEX are rich and the outlined performance comparisons suggest that CODEX offers highly competitive performance across diverse applications.

CODEX builds on counterfactual reasoning and implements different perturbations via distinct model representations,

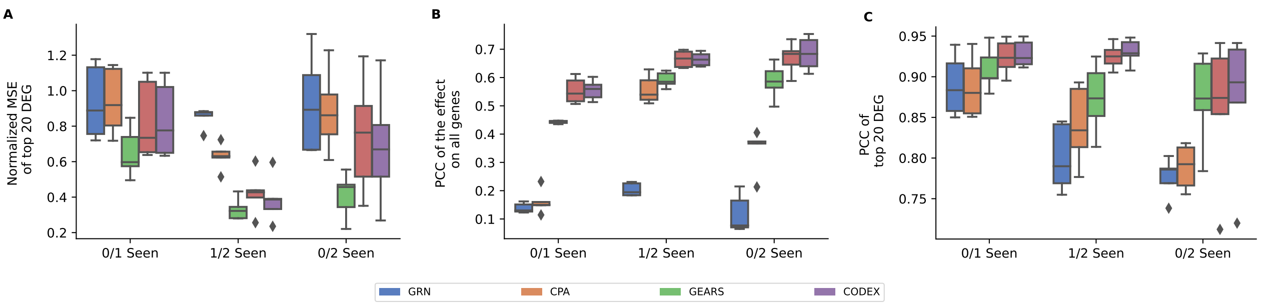

Fig. 7. Reconstruction performance of unseen perturbations for varying degrees of difficulty. The inferred effect of perturbations is evaluated for a single missing perturbation (0/1 seen), a missing perturbation in a pair (1/2 seen), and two missing perturbations in a pair (0/2 seen) on the Norman *et al.* (2019) data.

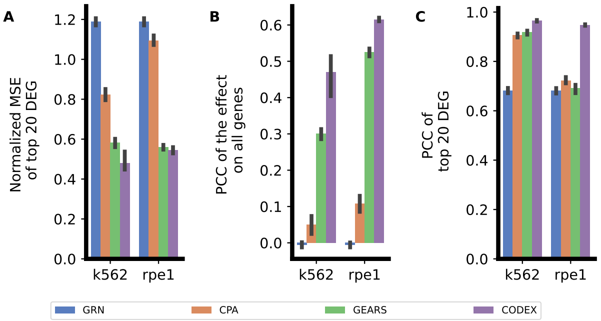

Fig. 8. Reconstruction performance for an unseen single perturbation on single cells from K562 and RPE-1 cell lines (Replogle *et al.*, 2022).

while most state-of-the-art approaches include perturbations as distinct variables (Lotfollahi *et al.*, 2023; Roohani *et al.*, 2023) or represent them using chemical embeddings (Preuer *et al.*, 2018; Kuru *et al.*, 2021; El Khili *et al.*, 2023). Causal modeling of representations increases model complexity, but has the benefit that highly complex downstream effects can be captured through the model architecture. This might be particularly relevant for the prediction of complex phenotypes, as our empirical results for the prediction of perturbed single-cell transcriptomes suggest. To enable out-of-distribution predictions for unseen drug combinations, different perturbation branches are combined. This has two advantages. First, subsequent network layers can account for potential non-linearities. Those were repeatedly shown to be key to the outlined prediction tasks as supported by our comparison to the linear CODEX baseline (lin-CODEX). Second, and most importantly, CODEX can learn the individual perturbation from combinations of perturbations. Given the combinatorial complexity of high-throughput perturbation screens, it is not tractable to comprehensively explore the space of perturbations and their downstream effects experimentally. However, CODEX makes use of redundancy, and might further improve extrapolation performance as perturbations are captured in more and more diverse combinations, not even limited to pairs of perturbations. This will be crucial to maximize the use of upcoming, potentially more comprehensive perturbation screens.

CODEX has a number of limitations. First, the main issue that afflicts all prediction algorithms of such a kind is that *in vitro* cell line experiments provide only a very limited picture of the mechanisms taking place *in vivo*. It will be key to further consider the *in vivo* model transfer. Multiple approaches were already suggested, comprising Velodrome (Sharifi-Noghabi *et al.*, 2021) and CODE-AE (He *et al.*, 2022). However, they mainly rely on a measure of distributional similarity and do not address that biological processes between human and cell lines differ, with the former comprising, e.g., complex cellular interactions taking place in the tumor microenvironment. Second, nowadays perturbation experiments are destructive, meaning that we never observe an individual cell before and after the intervention. Observing the latter might substantially deepen our understanding of molecular downstream effects. In this direction, e.g., Bunne *et al.* (2023) and Dong *et al.* (2023), attempt to identify counterfactual cell pairs using optimal transport. However, a combination of those methods with CODEX requires future research, addressing, e.g., potential biases from cell-pair selection.

In summary, CODEX provides a highly potent framework to extrapolate the space of interventions in high throughput perturbation experiments to unseen interventions and combinations thereof. Empirical results suggest that it substantially enhances out-of-distribution predictions and applies to diverse prediction tasks, suggesting rich applications in pharmacogenomics.

Data and availability

All data used in this article are publicly available. The Gene Expression Omnibus accession numbers to obtain the raw datasets are: Srivatsan *et al.* (2020) GSM4150378, Lotfollahi *et al.* (2023) GSE206741, Norman *et al.* (2019) GSE133344. The data from Replogle *et al.* (2022) are available from `https://doi.org/10.25452/figshare.plus.20022944` and the prepossessed drug-synergy data from `https://github.com/Emad-COMBINE-lab/MARSY/tree/main/data` (El Khili *et al.*, 2023). Further information and code to reproduce the experiments are provided in the CODEX repository at `https://github.com/sschrod/CODEX`.

Funding

The work of HUZ and MA was supported by the German Federal Ministry of Education and Research (BMBF) within the framework of the e:Med research and funding concept [01ZX1912A, 01ZX1912C]. SS and MA further acknowledge support by the BMBF [01KD2209D], and by

the Deutsche Forschungsgemeinschaft (DFG, German Research Foundation) [AL 2355/1-1 "Digital Tissue Deconvolution - Aus Einzelzelldaten lernen"]. TB and ACH were supported by the BMBF projects FAIrPaCT [01KD2208A]. TB was further supported by the BMBF projects PerMiCCion [01KD2101C] and MATCH [01KU1910A] and DFG TRR274 [408885537] and KFO5002 [426671079].

Conflict of Interest: none declared.

References

Al-Lazikani, B. *et al.* (2012). Combinatorial drug therapy for cancer in the post-genomic era. *Nature biotechnology*, **30**(7), 679–692.

Bliss, C. I. (1939). The toxicity of poisons applied jointly 1. *Annals of applied biology*, **26**(3), 585–615.

Bunne, C. *et al.* (2023). Learning single-cell perturbation responses using neural optimal transport. *Nature methods*, pages 1–10.

Bush, E. C. *et al.* (2017). Plate-seq for genome-wide regulatory network analysis of high-throughput screens. *Nature communications*, **8**(1), 105.

Consortium, G. O. (2004). The gene ontology (go) database and informatics resource. *Nucleic acids research*, **32**(suppl_1), D258–D261.

Costello, J. C. *et al.* (2014). A community effort to assess and improve drug sensitivity prediction algorithms. *Nature biotechnology*, **32**(12), 1202–1212.

Csermely, P. *et al.* (2013). Structure and dynamics of molecular networks: a novel paradigm of drug discovery: a comprehensive review. *Pharmacology & therapeutics*, **138**(3), 333–408.

Dong, M. *et al.* (2023). Causal identification of single-cell experimental perturbation effects with cinema-ot. *Nature Methods*, pages 1–11.

Douglass, E. F. *et al.* (2022). A community challenge for a pancancer drug mechanism of action inference from perturbational profile data. *Cell Reports Medicine*, **3**(1).

El Khili, M. R. *et al.* (2023). Marsy: a multitask deep-learning framework for prediction of drug combination synergy scores. *Bioinformatics*, **39**(4), btad177.

He, D. *et al.* (2022). A context-aware deconfounding autoencoder for robust prediction of personalized clinical drug response from cell-line compound screening. *Nature Machine Intelligence*, **4**(10), 879–892.

Iorio, F. *et al.* (2016). A landscape of pharmacogenomic interactions in cancer. *Cell*, **166**(3), 740–754.

Janizek, J. D. *et al.* (2018). Explainable machine learning prediction of synergistic drug combinations for precision cancer medicine. *BioRxiv*, page 331769.

Johansson, F. *et al.* (2016). Learning representations for counterfactual inference. In *International conference on machine learning*, pages 3020–3029. PMLR.

Kuru, H. I. *et al.* (2021). Matchmaker: a deep learning framework for drug synergy prediction. *IEEE/ACM transactions on computational biology and bioinformatics*, **19**(4), 2334–2344.

Ling, A. *et al.* (2018). More than fishing for a cure: The promises and pitfalls of high throughput cancer cell line screens. *Pharmacology & therapeutics*, **191**, 178–189.

Loewe, S. (1953). The problem of synergism and antagonism of combined drugs. *Arzneimittel-forschung*, **3**(6), 285–290.

Lotfollahi, M. *et al.* (2023). Predicting cellular responses to complex perturbations in high-throughput screens. *Molecular Systems Biology*, page e11517.

Norman, T. M. *et al.* (2019). Exploring genetic interaction manifolds constructed from rich single-cell phenotypes. *Science*, **365**(6455), 786–793.

Preuer, K. *et al.* (2018). Deepsynergy: predicting anti-cancer drug synergy with deep learning. *Bioinformatics*, **34**(9), 1538–1546.

Reinhold, W. C. *et al.* (2012). Cellminer: a web-based suite of genomic and pharmacologic tools to explore transcript and drug patterns in the nci-60 cell line set. *Cancer research*, **72**(14), 3499–3511.

Replogle, J. M. *et al.* (2022). Mapping information-rich genotype-phenotype landscapes with genome-scale perturb-seq. *Cell*, **185**(14), 2559–2575.

Roohani, Y. *et al.* (2023). Predicting transcriptional outcomes of novel multigene perturbations with gears. *Nature Biotechnology*, pages 1–9.

Rubin, D. B. (1974). Estimating causal effects of treatments in randomized and nonrandomized studies. *Journal of educational Psychology*, **66**(5), 688.

Schrod, S. *et al.* (2022). Bites: balanced individual treatment effect for survival data. *Bioinformatics*, **38**(Supplement 1), i60–i67.

Schrod, S. *et al.* (2023). Adversarial distribution balancing for counterfactual reasoning. *arXiv preprint arXiv:2311.16616*.

Shalit, U. *et al.* (2017). Estimating individual treatment effect: generalization bounds and algorithms. In *International conference on machine learning*, pages 3076–3085. PMLR.

Sharifi-Noghabi, H. *et al.* (2021). Out-of-distribution generalization from labelled and unlabelled gene expression data for drug response prediction. *Nature Machine Intelligence*, **3**(11), 962–972.

Srivatsan, S. R. *et al.* (2020). Massively multiplex chemical transcriptomics at single-cell resolution. *Science*, **367**(6473), 45–51.

Subramanian, A. *et al.* (2017). A next generation connectivity map: L1000 platform and the first 1,000,000 profiles. *Cell*, **171**(6), 1437–1452.

Yadav, B. *et al.* (2015). Searching for drug synergy in complex dose–response landscapes using an interaction potency model. *Computational and structural biotechnology journal*, **13**, 504–513.

Yao, L. *et al.* (2018). Representation learning for treatment effect estimation from observational data. *Advances in neural information processing systems*, **31**.

Ye, C. *et al.* (2018). Drug-seq for miniaturized high-throughput transcriptome profiling in drug discovery. *Nature communications*, **9**(1), 4307.

Yoon, J. *et al.* (2018). Ganite: Estimation of individualized treatment effects using generative adversarial nets. In *International conference on learning representations*.

Zagidullin, B. *et al.* (2019). Drugcomb: an integrative cancer drug combination data portal. *Nucleic acids research*, **47**(W1), W43–W51.
