# Supplementary material for: CODEX: COunterfactual Deep learning for the in silico EXploration of cancer cell line perturbations
Source: Bioinformatics. 2024 Jun 28;40(Suppl 1):i91–9. doi: 10.1093/bioinformatics/btae261 (PMC11211812; doi:10.1093/bioinformatics/btae261)
Supplement: btae261_Supplementary_Data [file btae261_supplementary_data.zip › btae261_Supplementary_Data/Schrod.68.sup.1.pdf]

Supplementary Material: *COunterfactual Deep learning for the in-silico EXploration of cancer cell line perturbations*

S1 Implementation details

CODEX is implemented using the PyTorch machine learning library. It uses a dense feed-forward architecture in combination with ReLU activation functions and Dropout layers. Further, it uses early-stopping to prevent overfitting and the minimal/maximal validation score is used to select the best performing model. Our implementation of CODEX is publicly available at `https://github.com/sschrod/CODEX`. All experiments were run on a cluster with 8 Nvidia A100 GPUs, 256 CPU cores and 512GB of RAM. Nevertheless, running the reconstruction experiments is computationally feasible for almost any modern GPU with CODEX requiring less than 5GB of GPU memory. Note that the drug-synergy experiments require more GPU memory due to the large treatment specific layers.

Table S1: CODEX hyper-parameter ranges for the drug-synergy experiment.

Parameter	Range
Dim. layers	$\{[4096, 2048, 1024, 512], [2048, 1024, 512, 256]\}$
Learning Rate $\{0.0001\}$ Batch Size	$\{4096, 1024\}$
Dropout	$\{0.2, 0.4\}$
Weight Decay	$\{0.001, 0.01, 0.1, 1\}$

S2 The drug-synergy experiment

The hyper-parameter ranges used for the drug-synergy experiment are listed in Table S1. We performed an extensive grid search, covering all possible combinations of the listed parameters. Following El Khili et al. [2023] and Preuer et al. [2018], we used a large architecture with many of nodes. The layers are split into two encoder layers, one treatment specific layer and one decoder layer, according to the "Dim. layers" parameters outlined in Table S1. The decoder is further connected to a single outcome node, representing the synergy score.

All models were trained until the validation MSE (10% of the training data) did not increase for 50 consecutive epochs. Finally, the best model was selected based on minimal validation MSE.

In addition to the results for the ZIP synergy score reported in the main manuscript, Table S2 shows the performance obtained for the S_{mean} synergy score (following El Khili et al. [2023]).

Table S2: Mean performance of S_{mean} synergy prediction on stratified 5-fold cross validation holding out specific drug pairs for testing.

Model	Setting 1			Setting2		
	SCC	PCC	RMSE	SCC	PCC	RMSE
CODEX	0.820 (±.003)	0.859 (±.003)	8.59 (±.10)	0.800 (±.005)	0.838 (±.008)	9.13 (±.37)
MARSY	0.836 (±.002)	0.864 (±.003)	8.42 (±.09)	0.809 (±.007)	0.841 (±.011)	9.06 (±.45)
MatchMaker	0.810 (±.003)	0.840 (±.005)	9.84 (±.23)	0.788 (±.009)	0.816 (±.015)	10.34 (±.46)
TreeCombo (XGBoost)	0.817 (±.002)	0.852 (±.002)	8.77 (±.06)	0.775 (±.010)	0.815 (±.011)	9.69 (±.37)
DeepSynergy	0.803 (±.003)	0.843 (±.002)	9.14 (±.12)	0.762 (±.004)	0.804 (±.011)	10.17 (±.42)

Table S3: CODEX hyper-parameter ranges for the drug-dose and Combosciplex experiment.

Parameter	Range
Dim. layers	$\{[1024, 512, 256], [512, 256, 128]\}$
Learning Rate $\{0.001, 0.0001\}$ Batch Size	$\{1024, 2048\}$
Dropout	$\{0.0, 0.2\}$
Weight Decay	$\{0.001, 0.00001, 0.0000001\}$

S3 Drug-dose and Combosciplex experiments

Both, the drug-dose and Combosciplex experiments use the same set of hyper-parameters outlined in Table S3. Here, the dimension of the layers describes the 3 layer encoder, the latent representations consist of a single layer. The size of the last decoder layer and the decoder is given by the same 3 layer structure as the encoder, but in reversed order. For all reconstruction experiments, the decoder maps to a final outcome consisting of the mean and the variance of the post-perturbation data (the outcome has twice the size of the input features). Accordingly, the model was optimized using the Gaussian Negative-log-likelihood. Similar to the previous experiment, we performed a grid search covering all possible parameter combinations and the best models were selected based on maximal reconstruction performance in terms of the validation coefficient of determination R^2 on the top 50 DEGs. For both experiments, we used 25% of the training data for validation and a patience of 50 epochs.

In addition to the individual dose-response curves, we report the median reconstruction performance for all doses (on validation and ood data) in Figure S1a and for the unseen dose (ood) in Figure S1b.

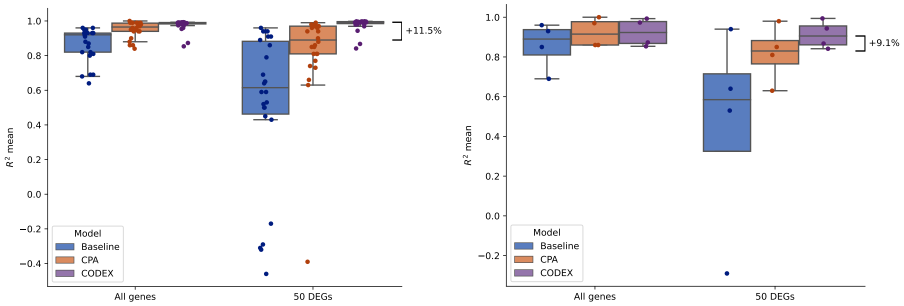

(a) Ood dosages.

(b) Test and ood dosages.

Figure S1: Box plot for the reconstruction performance on Sciplex2 data.

Further, Table S4 shows all treatment combinations present in the Combosciplex data, with control samples highlighted in blue and ood combinations in red. Figure S2 shows the median reconstruction performance for the left out combinations.

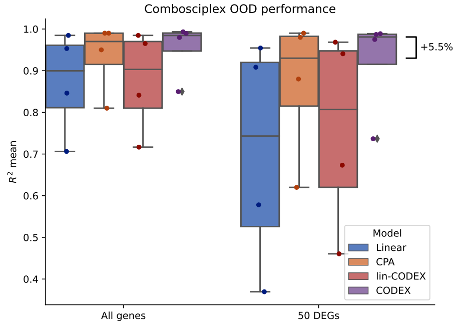

Figure S2: Box plot of the reconstruction performance of the four unobserved treatment combinations on Combosciplex data.

S4 Gene-perturbation experiments

The experimental design of perturbation experiment on the Norman et al. [2019] follows the design of the drug-dose and Combosciplex experiments. The used hyper-parameters are listed in Table S5. In contrast to previous experiments, we evaluated the perturbation experiments with respect to PCCs and reconstruction MSE (following Roohani et al. [2023]). Hence, we selected the best performing model based on the minimal validation MSE for the top 20 DEGs.

In addition to the main experiments on Norman et al. [2019] data, we repeated the experiment of Lotfollahi et al. [2023]. Accordingly, we used 13

Table S4: List of all Combosciplex perturbation combinations, ood combinations are marked in red and control samples in blue

Compound 1	Compound 2	Number of Cells
Dacinostat	PCI-34051	3302
SRT3025	Cediranib	3017
Givinostat	Cediranib	2785
DMSO	SRT2104	2757
Givinostat	Curcumin	2739
Givinostat	Sorafenib	2736
Givinostat	Carmofur	2694
Givinostat	crizotinib	2666
Givinostat	dasatinib	2421
Givinostat	SRT2104	2357
DMSO	dasatinib	2343
Givinostat	SRT1720	2265
panobinostat	Curcumin	2244
Cediranib	PCI-34501	2166
panobinostat	Sorafenib	2013
panobinostat	SRT2104	1974
panobinostat	dasatinib	1956
Dacinostat	Danusertib	1941
panobinostat	SRT3025	1890
DMSO	Dacinostat	1869
panobinostat	SRT1720	1828
panobinostat	PCI-34051	1814
DMSO	Givinostat	1685
panobinostat	crizotinib	1643
DMSO	panobinostat	1578
DMSO	DMSO	1451
Givinostat	Tanespimycin	1314
Dacinostat	dasatinib	1231
panobinostat	Alvespimycin	996
DMSO	Alvespimycin	758
SRT2104	Alvespimycin	520
Alvespimycin	Pirarubicin	477
		63430

Table S5: CODEX hyper-parameter ranges for the perturbation experiments.

Parameter	Range
Dim. layers	$\{[1024, 512, 256], [512, 128, 64]\}$
Learning Rate $\{0.001, 0.0001\}$ Batch Size	$\{1024, 512, 256\}$
Dropout	$\{0.1, 0.2, 0.4\}$
Weight Decay	$\{0.00001, 0.000001, 0.0000001\}$

train/test splits with $\sim$ 10 perturbation combinations left out in each split, covering all of the 131 two gene perturbations. Figure S4 shows the R^2 reconstruction performance for all genes and the top 50 DEGs, comparing CODEX to CPA Lotfollahi et al. [2023], to the linear baseline and to the random baseline.

Following the results on the Norman et al. [2019] data, we evaluated the Replogle et al. [2022] data for the set of parameters that was most frequently selected. Hence, we used the architecture with a decoder consisting of $[512, 128, 64]$ nodes, a learning rate of 0.001, a batch size of 256, a dropout rate of 0.1, and weight decay of 10^{-6}.

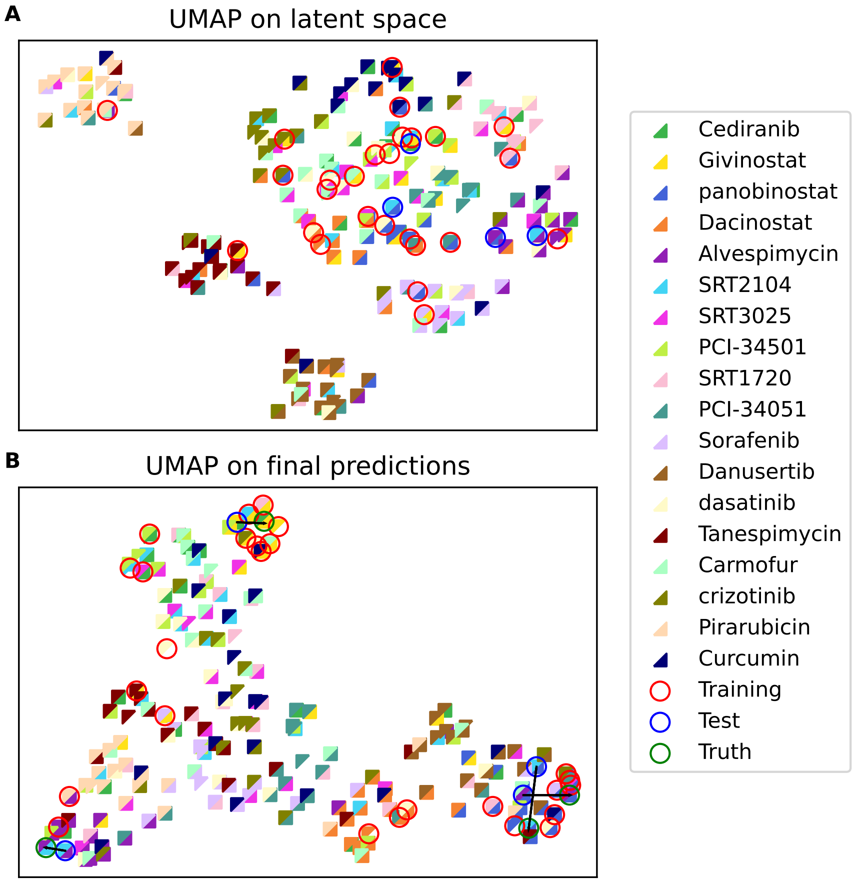

Figure S3: R^2 reconstruction performance of the mean post perturbation gene expression profiles for all genes (blue) and top 50 DEGs (orange) obtained for the four held out treatment combinations on the Combosciplex data (full resolution).

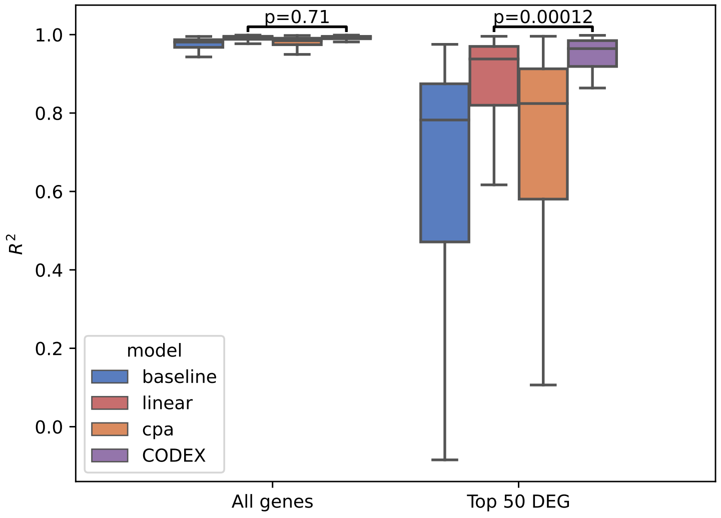

Figure S4: Reconstruction performance in terms of R^2 for all genes (left) and the top 50 DEGs (right) in the experimental setting of Lotfollahi et al. [2023].

References

Mohamed Reda El Khili, Safyan Aman Memon, and Amin Emad. Marsy: a multitask deep-learning framework for prediction of drug combination synergy scores. *Bioinformatics*, 39(4):btad177, 2023.

Mohammad Lotfollahi, Anna Klimovskaia Susmelj, Carlo De Donno, Leon Hetzel, Yuge Ji, Ignacio L Ibarra, Sanjay R Srivatsan, Mohsen Naghipourfar, Riza M Daza, Beth Martin, et al. Predicting cellular responses to complex perturbations in high-throughput screens. *Molecular Systems Biology*, page e11517, 2023.

Thomas M Norman, Max A Horlbeck, Joseph M Replogle, Alex Y Ge, Albert Xu, Marco Jost, Luke A Gilbert, and Jonathan S Weissman. Exploring genetic interaction manifolds constructed from rich single-cell phenotypes. *Science*, 365(6455):786–793, 2019.

Kristina Preuer, Richard PI Lewis, Sepp Hochreiter, Andreas Bender, Krishna C Bulusu, and Günter Klambauer. Deepsynergy: predicting anti-cancer drug synergy with deep learning. *Bioinformatics*, 34(9):1538–1546, 2018.

Joseph M Replogle, Reuben A Saunders, Angela N Pogson, Jeffrey A Hussmann, Alexander Lenail, Alina Guna, Lauren Mascibroda, Eric J Wagner, Karen Adelman, Gila Lithwick-Yanai, et al. Mapping information-rich genotype-phenotype landscapes with genome-scale perturb-seq. *Cell*, 185(14):2559–2575, 2022.

Yusuf Roohani, Kexin Huang, and Jure Leskovec. Predicting transcriptional outcomes of novel multigene perturbations with gears. *Nature Biotechnology*, pages 1–9, 2023.
